# LATE‐NC IN THE VERY ELDERLY– THE POPULATION‐BASED VANTAA 85+ ‐STUD

**DOI:** 10.1002/alz.094649

**Published:** 2025-01-09

**Authors:** Elizaveta Mikhailenko, Kia Colangelo, Jarno Tuimala, Mia Kero, Sara Emilia Savola, Anna Raunio, Eloise H Kok, Maarit Tanskanen, Mira Mäkelä, Henri Puttonen, Mikko I. Mäyränpää, Darshan Kumar, Karri Kaivola, Anders Paetau, Pentti Tienari, Tuomo Polvikoski, Liisa Myllykangas

**Affiliations:** ^1^ University of Helsinki, Helsinki, Uusimaa Finland; ^2^ Finnish Tax Administration, Helsinki, Uusimaa Finland; ^3^ HUS Diagnostic Center, Helsinki, Uusimaa Finland; ^4^ Tampere University, Tampere, Pirkanmaa Finland; ^5^ University of Helsinki, Helsinki Finland; ^6^ Helsinki University Central Hospital, Helsinki Finland; ^7^ Aiforia Technologies Plc., Helsinki, Uusimaa Finland; ^8^ Helsinki University Hospital, Helsinki, Uusimaa Finland; ^9^ Newcastle University, Newcastle upon Tyne, Norteastern England United Kingdom

## Abstract

**Background:**

Population‐based cohort studies play a crucial role in unraveling the underlying causes of dementia among older individuals. While previous research has indicated an increase in limbic‐predominant age‐related TDP‐43 encephalopathy neuropathological change (LATE‐NC) with age, only limited investigations have delved into this phenomenon within a population‐based context. In this study, we examined the prevalence of LATE‐NC and its correlations with other brain pathologies and cognitive function in individuals aged > 85 years.

**Method:**

Evaluation of the Vantaa 85+ study cohort, comprising 601 individuals aged >85 years residing in Vantaa, Finland in 1991, formed the basis of our investigation. Neuropathological assessments were conducted on 304 subjects (51%), with LATE‐NC staging feasible for 295. Dementia status and Mini‐Mental State Examination scores were established during baseline and three follow‐up assessments spanning from 1994 to 1999. LATE‐NC staging relied on TDP‐43 immunohistochemistry, following recently updated guidelines. Digital evaluation of arteriolosclerosis involved calculating the average sclerotic index of five randomly selected small arterioles in the amygdala, hippocampal regions, and frontal white matter. Fisher’s exact test, along with linear and logistic regression models, were employed to analyse associations between LATE‐NC, arteriolosclerosis, previously identified neuropathological variables (such as Alzheimer’s disease neuropathological change, Lewy‐related pathology, hippocampal sclerosis, and cerebral amyloid angiopathy), and cognitive metrics.

**Result:**

Among the 295 subjects, LATE‐NC was present in 189 (64%), with stage 2 being the most prevalent (29%) followed by stage 3 (13%), while stages 1a, 1b, and 1c were less frequent (10%, 5%, and 8%, respectively). Stages 1a (P< 0.01), 2 (P< 0.001), and 3 (P< 0.001) were significantly associated with dementia and lower Mini‐Mental State Examination scores. LATE‐NC exhibited strong associations with Alzheimer’s disease neuropathological change (P< 0.001), hippocampal sclerosis (P< 0.001), diffuse neocortical Lewy‐related pathology type (P< 0.001), and amygdala arteriolosclerosis (P< 0.006). Across all six multivariate models, LATE‐NC emerged as one of the most robust independent predictors of dementia. Notably, LATE‐NC commonly co‐occurred with other neuropathological changes, with only a negligible percentage (<0.5%) of cases attributing dementia solely to LATE‐NC.

**Conclusion:**

This population‐based inquiry underscores the significant role of LATE‐NC as an independent determinant of dementia within the general late‐life population.

